# The Use of Digital Microscopy to Compare the Thicknesses of Normal Corneas and *Ex Vivo* Rejected Corneal Grafts with a Focus on the Descemet's Membrane

**DOI:** 10.1155/2019/8283175

**Published:** 2019-11-15

**Authors:** Taíse Tognon, Sabrina Bergeron, Christina Mastromonaco, Kleyton Barella, Adriano Pasqualotti, Laura Nunez, Francisco Murta, Luciene Barbosa de Sousa, Mauro Campos, Miguel Noel Nascentes Burnier

**Affiliations:** ^1^Federal University of São Paulo, MUHC–McGill University Ocular Pathology & Translational Research Laboratory, Penido Burnier Institute, Campinas, Brazil; ^2^MUHC–McGill University Ocular Pathology & Translational Research Laboratory, Montreal, Canada; ^3^Penido Burnier Institute, Campinas, Brazil; ^4^University of Passo Fundo, Lisbon University, Passo Fundo, Brazil; ^5^Université de Montréal, MUHC–McGill University Ocular Pathology & Translational Research Laboratory, Montreal, Canada; ^6^Lisbon University, The MUHC–McGill University Ocular Pathology & Translational Research Laboratory, Lisbon, Portugal; ^7^Federal University of São Paulo, Goiás Eye Bank, São Paulo, Brazil; ^8^Federal University of São Paulo, São Paulo, Brazil; ^9^MUHC–McGill University Ocular Pathology & Translational Research Laboratory, Federal University of São Paulo, Montreal, Canada

## Abstract

**Objective:**

To compare the thickness of corneal layers, specifically the Descemet's membrane (DM), in normal corneas and in failed grafts due to rejection (FGRs) using the digital histopathology and to propose a model for the measurement of corneal layers using this method.

**Methods:**

This is a prospective, cross-sectional study performed at the MUHC-McGill University Ocular Pathology & Translational Research Laboratory (McGill University, Montreal, Canada). Histopathological sections of 25 normal human corneas and 40 FGRs were fully digitalized and examined. Inclusion criteria: samples diagnosed as normal corneas or FGRs, from patients older than 18 years of age. Exclusion criteria: histopathological sections without adequate tissue or missing epidemiological information. For each sample, the thicknesses of the epithelium, stroma, and DM were acquired. From a perpendicular plane of reference, two central measurements and two nasal and two temporal peripheral measurements were obtained.

**Results:**

There were differences between the normal and FGR groups in the mean central thickness of the epithelium (*p* < 0.001), the nasal and temporal stromal regions (*p* < 0.001), and of the DM in the nasal and temporal regions (*p* < 0.001). Compared with the extremities of the sample (nasal and temporal), the mean thickness of the DM in normal corneas was lower in the central region (*p* < 0.001), and this difference was not found in the FGR group.

**Conclusions:**

Normal corneas have a thinner epithelium in the central region than the FGR group. In addition, the stroma and DM thicknesses of the nasal and temporal periphery were significantly higher in normal corneas than in those from the FGR group. The digital microscopy protocol applied in this study may be useful for further research studies regarding cornea and other tissues.

## 1. Introduction

The cornea has long been the target of surgical procedures and grafting attempts with the goal of restoring vision. Contemporary surgical techniques for corneal transplantation have accrued from innumerable ideas and perseverance over the centuries [1], and interest in this theme continues to grow.

The main cause of corneal transplant failures is rejection. Studies have shown that at least 30% of eyes undergoing penetrating keratoplasty experienced at least one rejection episode in their lifetime, and approximately 5–7% of these rejections led to eventual failure [2–5].

Early diagnosis of corneal graft rejection and/or failure has been a challenge for many researchers, and histological *in vivo* and *ex vivo* studies have demonstrated progress on this issue [6–10]. However, studies regarding the Descemet's membrane (DM, the true basal membrane of the cornea) remain scarce.

With the advent of new high-resolution corneal computed tomography (HR-OCT) scans, other researchers have investigated methods for early detection of corneal rejection and/or failure, performing detailed analysis of corneal thickness [11–16]. Digital imaging technologies has also been revolutionizing medicine through the initiation of histopathology scanners and processors, making the process faster and the possibility of using new analysis tools [17, 18].

Therefore, the aim of this study was to compare the thickness of the corneal layers, specifically the Descemet's membrane (DM), using digital pathology, in normal corneas and failed corneal grafts due to rejection. The Descemet's membrane was of specific interest due to the strong evidence of thickening of the basement membranes in transplants of solid organs and its correlation with rejection and failure [13, 19–22]. Another proposal of this study is to develop a protocol for the measurement of corneal layers using digital microscopy.

## 2. Materials and Methods

This is a prospective, cross-sectional study performed at the MUHC-McGill University Ocular Pathology & Translational Research Laboratory (Montreal, QC, Canada).

This research was part of a project approved by the Ethics Committee of Federal University of São Paulo (opinion number 1.642.065), conducted in accordance with the guiding principles of the Declaration of Helsinki.

### 2.1. Samples

Human corneal specimens were obtained from whole-globes from the Minnesota Eye Bank, US, Alabama Eye Bank, US, and from the laboratory's digital pathology database.

The inclusion criteria were as follows:

- Histopathological sections with clinical diagnoses of normal cornea or failed corneal grafts due to rejection,
- Tissue samples from patients older than 18 years of age.

The exclusion criteria were as follows:

- Inadequately prepared histopathological sections or those with corneal layers that could not be completely visualized by microscopy,
- Incomplete records.

Normal corneas were defined as tissues from patients who had not previously undergone intraocular surgeries and had no ocular diseases. Failed corneal grafts due to rejection included patients with this primary diagnosis; however, other diseases could be associated with this diagnosis.

### 2.2. Histopathological Preparation

Enucleation samples were initially fixed by immersion in 10% formalin. Subsequently, the anterior segment was isolated, and the nasal, temporal, superior, and inferior regions were marked.

All tissues were processed and embedded in paraffin as per routine histopathology protocol. Tissue vertical sections measuring 5 *μ*m of thickness were obtained, and the slides from the central region of the blocks were stained with hematoxilin-eosin (H&E) and periodic acid-Schiff (PAS) following a conventional staining protocol.

### 2.3. Scanning of the Slides

Whole-slide images were obtained from the glass slides using a Philips Ultra Fast Scanner 1.6 RA® (Philips Digital Pathology; Best, North Brabant, Netherlands), with 40x magnification and resolution of 0.25 *μ*m per pixel.

### 2.4. Corneal Layer Measurements

Measurements of the corneal layers were obtained using the *IntelliSite Pathology Solution*® “ruler” tool from the scanner.

**Figure 1:**
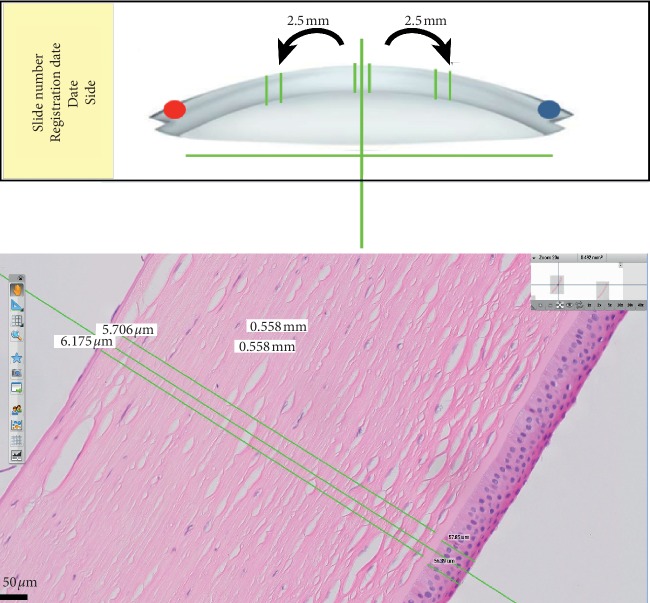
Schematic drawing of the references used to obtain corneal measurements using digital microscopy software (above) and detail of measurements in the corneal samples, hematoxilin-eosin (H&E) staining, 20x magnification (below).

For this measurement, a line was drawn parallel to the fixation plane of the corneal tissue on the slide and another line perpendicular (exact 90° angle) at 1x magnification, which was used as a reference for all of the measurements (Figure 1).

The central region was defined as that located geographically in the center of the sample, and the peripheral regions were defined as 2.5 millimeters (mm) distant from the center point toward the sides of the sample (Figure 1). The peripheral regions were determined by the size of the corneal buttons, the smallest diameter of which was 6 mm.

The unit of measurement was automatically calculated by the ruler, in millimeter (mm) when using a 10x magnification or in micrometer (*μ*m) when using a 20x or 40x magnification.

For each sample, two equidistant measurements in the nasal tissue, two equidistant measurements in the temporal periphery, and two measurements in the central region of the tissue were recorded for all the previously cited layers (Figure 1).

The measurements of the epithelium and the DM were determined using the 40x objective of the digital image, while the measurements of the stroma and total corneal thickness were initially determined using the 10x magnification and then were adjusted using the 20x and 40x magnification.

It is worth noting that the structures of interest are not automatically located by the software; instead, the user is responsible for identifying the areas to be studied.

In the measurements using manual optical microscopy only, an Olympus EX41® microscope (Olympus Corporation, Shenzhen, Guangdong, Japan) was used considering a 10x, 20x, and 40x objective magnification.

### 2.5. Internal Validation: Comparison of the Measurements Obtained Manually Only and by Digital Microscopy

For the internal validation of the study, the thickness was measured in 10 randomly chosen sections from rejected/failed grafts and 10 sections from normal corneas previously stained with PAS. Comparisons were made between the values obtained with the manual method *versus* the digital microscopy method. PAS staining was chosen to highlight the Descemet's membrane and the basement membrane of the epithelium from other structures. To avoid extra costs, this stain was used for internal validation only.

Initially, 10 samples from each group were randomly chosen to perform this internal validation. If the measures obtained did not have statistical power, the number of samples was gradually increased.

As an initial reference of the measurements, the corneal central region was demarcated, in which the interface of the epithelium in the central region was marked with a fountain pen for use as a guide. All of the measurements were performed by 2 previously trained doctors with experience in ocular pathology: an ophthalmologist familiar with digital microscopy (TT) and a pathologist familiar with optical microscopy (MB).

### 2.6. Data Collection

The following information was collected from normal corneas and corneas from FGRs: slide registration number; age; sex; side (left vs. right); previous eye disease; and thickness measurements obtained digitally from the central, nasal, and temporal regions (two measurements in *μ*m or mm for each region) for the epithelium, stroma, and DM.

The information collected was compiled in Microsoft Office Excel® tables (Microsoft Corporation, Redmond, Washington, USA).

### 2.7. Statistical Analysis

The IBM SPSS Statistics Base 22.0® software (SPSS, Inc., Chicago, Illinois, USA) was used for descriptive and comparative analyses. Comparisons of two independent variables were performed using Student's *t*-test for continuous variables and chi-squared test for categorical variables. For multiple comparisons, ANOVA and Tukey's test were used. Levene's test was used to analyze homogeneity, and the Kolmogorov–Smirnov test was used to assess the equality of probability distributions. Statistical significance was considered when *p* < 0.05.

## 3. Results

The study included 25 normal *postmortem* corneas and 75 corneas from FGRs. Thirty-five of the FGRs were excluded from the study because they did not meet our tissue quality criteria.

Of the 25 normal corneas, 20 came from female patients and 5 from male patients; of the 40 corneas that failed due to rejection, 20 were from female patients and 20 from male patients.

Past medical history listed for these patients was as follows: pseudophakic bullous keratopathy (*n*=10); various corneal opacities (*n*=11); corneal dystrophy (*n*=1, unspecified); uveitis (*n*=1); keratoconus (*n*=6); and Fuchs's dystrophy (*n*=6). Moreover, three patients had histories of corneal herpes; one had Cogan syndrome; and one had iridocorneal endothelial syndrome.

For the patients in the FGR group, previous surgical interventions were as follows: penetrating keratoplasty (PK) (*n*=1), automated endothelial keratoplasty (DSAEK) (*n*=6), two penetrating keratoplasty (*n*=2), one PK plus one DSAEK (*n*=2), and three PKs (*n*=2). Lastly, two patients have had three previous transplants: one PK and two DSAEK. Patients who underwent previous endothelial keratoplasty did not present significant differences in the measurements of the corneal layers, compared with the other patients.

The age (mean ± standard deviation) of the patients whose corneas were normal was 81.8 ± 6.0 (range 71–95) years old and that of the patients whose corneas were from failed grafts was 66.2 ± 14.6 (range 31–88) years old, with a significant difference between the groups (*p* < 0.001).

Considering the sampling of normal corneas, two measurements were obtained in the central region of the epithelium, stroma, and DM; two measurements in the nasal region of the epithelium, stroma, and DM of each of the 25 corneas; and two measurements in the temporal region of the epithelium, stroma, and DM. For the 40 corneas from FGRs, the same measurement protocol was applied.

It is important to emphasize that, when the statistical tests were applied, the normality assumption was confirmed; thus, the sample was reliable for analysis.

Table 1 shows the results of the descriptive variables analyzed and the mean corneal thickness measurements of the central, nasal, and temporal epithelial, stromal, and DM regions of normal corneas and corneas from FGRs. It should be noted that all thickness measurements described in this table were obtained from the digitalized samples.

With respect to thickness measurements, there was a difference between the normal and FGRS in the central measurement of the epithelium (*p* < 0.001), in the nasal (*p* < 0.001) and temporal stroma regions, and in the nasal (*p* < 0.001) and temporal (*p* < 0.001) regions in the DM.

Table 2 summarizes the comparison between the mean thicknesses of the different regions of the DM within the same group (normal corneas × FRGFs) by digital microscopy. In normal corneas, the central region of the DM presented a lower mean thickness than the peripheral regions (*p* < 0.001), while in failed graft samples, this difference was not observed.

Tables 3 and 4 describe the results of internal validation of this study, obtained from compared measurements of normal corneas and FRGs using manual microscopy and digital microscopy. There was no significant difference between the measurements obtained with these two methods.

## 4. Discussion

The basement membrane is a structure that could potentially indicate signs of rejection and survival of grafts, according to studies in other organs [19, 21, 23]. There is strong evidence in the literature on solid organ transplants showing that measurement of the basement membrane thickness of allogeneic grafts in optical and electron microscopy studies can be used to detect rejection [13, 19–22, 24]. In addition, the DM has always been considered a true corneal basement membrane, which was why our research focused on the DM [25].

To detect these signs in kidney, liver, and lung transplants, invasive biopsies are necessary [13]. In contrast to these organs, the cornea is a transparent and avascular organ [13]. Based on this fact, some authors have aimed to determine the thickness of the DM using HR-OCT [13]. These authors performed an *in vivo* imaging study using HR-OCT of transplanted corneas, comparing them with normal controls. They measured the central corneal thickness and compared it with measurement of the DM/endothelium complex, creating an index and argued that measurement of the thickness of the DM/endothelium complex, as well as the index proposed for them, is a more accurate indicator of graft health than the total central corneal thickness [13].

Optical coherence tomographers, even the latest technology ones, do not yet have sufficient resolution to visually distinguish the DM from the endothelium, which could be a confounding factor in these *in vivo* studies involving HR-OCT since they can, for example, confuse images of endothelial edema with DM thickening.

Another study argued that the increased thickness of the DM/endothelium complex is due to thickening of the DM, based on an *ex vivo*, retrospective, histopathological study involving 54 corneas [23]. There was no difference in the total thickness of the corneas between the studied groups in the central region, but there were differences in the DM [23]. A limitation of this study was that the endothelium was studied in all normal corneas, whereas the same was not true for tissue samples of rejected grafts since they lack them.

According to Shousha et al., the DM becomes thicker over time [26]. In the analyses presented here, there was a difference in age between the groups; in the group with failed grafts, the mean age of patients was lower than that of patients with normal corneas. However, we did not have access in our study to the ages of the grafted corneal donors at the time that they were obtained. Therefore, many studies investigating the DM might have a confounding *bias* related to the age and donor corneas; perhaps, the donor was older, and thus their cornea was thicker or *vice versa*. In addition, it is not known whether the DM continues to thicken after transplantation similar to a normal cornea over time, whether this process stabilizes, or whether DM thickening is more intense due to surgical trauma (without a rejection episode, only as a result of the manipulation or inflammation inherent to the procedure).

These hypotheses can also justify the results found in the analysis of DM regions between donor corneas and failed corneas due to rejection: there was no significant difference between the measurements of the central region. The question of recipient age and the cornea donor could justify this finding, as well as injury at the time of surgery or the rejection reaction itself (which also consists of tissue injury). Some researchers have argued that the DM can thicken beyond normal when injured [13, 26].

Our findings differed from previous studies suggesting that the DM/endothelium complex thickens in the central region in actively rejecting corneas and that this thickening is due to the DM [13, 23]. It is noteworthy that one of these studies failed to separate the endothelial layer from the DM in its measurements, and the other could have been affected by problems during the histological preparation.

In the peripheral region of the DM, there was a difference between the groups studied. In the FRG group, the comparison of central DM thicknesses with nasal and temporal thicknesses did not reach statistical significance. To date, this was the first research to study regions other than the central corneal regions. Our results may be explained by the fact that, during the rejection process, the corneal periphery does not thicken but instead narrows due to the immunological reactions start there. As the DM is produced by peripheral endothelial corneal cells [25], the membrane has a tendency to retract following a trauma or insult [27]. Scarring that can occur after a chronic inflammatory process may also affect the shape of the cornea, leading to thinning [28]. According to Coster, scar tissue contracts with time, and this contraction may result in an area of depression in the cornea [28]. These hypotheses are in support of our findings.

Another possibility to consider is that the DM might not behave as other basal membranes of vascularized organs, such as kidneys, liver, and lungs, in the presence of an active immune process, such as rejection, because the corneal physiology differs greatly from the other organs of the human body. Perhaps the ideal location for the study of corneal rejections is not to look at the central region but instead at the periphery of the corneal button. It is also important to note that the tissues can be affected by different factors at the time of preparation of the samples. Tissue manipulation during surgery, formalin fixation, paraffin embedment, and storage methods are conditions that impact the quality of the histological specimen [29, 30], which could limit the improvement of histopathological studies.

Although measurements were obtained from the epithelial, stromal, and total corneal regions, some measurements were not considered if the tissue integrity was altered during removal, fixation, and repair of the tissue. As well known, the epithelium is especially subject to problems in histological preparation. Another artifact to be considered is stromal edema: corneal edema is underestimated on histopathological sections because of dehydration steps required to prepare the tissue for histopathological analysis [23]. Artifacts during histological preparation and structural alteration phenomena are less likely to occur with DM; hence, this corneal layer is preferred to be studied in the majority of the researches.

Moreover, it is important to highlight some differences described by authors in normal corneas and rejected corneas considering the stroma only. In the normal cornea, the collagen fibrils form approximately 300 distinct lamellae, each covering the entire area of the cornea and running parallel to the surface [28]. Transparency of the cornea is attributed to the extremely regular spacing of the collagen fibrils separated by glycosaminoglycans; the latter being responsible to maintain a uniform hydration of the stroma [28]. The concentrations and ratios of proteoglycans vary from anterior to posterior, and the posterior stroma is “wetter” than the anterior [31]. The lamellae of the anterior stroma are short, narrow sheets with extensive interweaving between layers, whereas the posterior stroma has long, wide, thick lamellae extending from limbus to limbus with minimal interlamellar connections [31].

The so-called “basket-weave” configuration of the corneal stroma can be lost in rejected corneas. In the acute phase, intense inflammation results in the recruitment of leukocytes, which are inflammatory cells containing lysozymes enzymes capable of disaggregating the cornea, causing tissue loss or stromal lysis [28].

Chronic inflammation of the cornea may follow acute inflammation if the initiating factor persists or may occur as a primary condition without an identifiable acute phase [28]. At a clinical level, chronic corneal inflammation is characterized by stromal edema and cellular infiltration, scarring, and neovascularization and at a histological level, and chronicity is characterized by a change in the inflammatory cell population [28]. There is also “budding” of limbal vessels, neovascularisation and lymphangiogenis, and the proliferation and migration of fibroblasts followed by fibrosis and creation of matrix materials [28]. The majority of these processes start in periphery of the cornea, and this could be why some differences were found in nasal and temporal regions between the two groups studied.

Another important factor to consider is the type of rejection encountered; in stromal rejection, the stroma may become necrotic, tuned, and disaggregated; or it may become swollen, as in the endothelial type of rejection [31]. In our study, it was not possible to obtain information regarding the type of rejection; this may be an interesting venue for future studies.

Notably, on average, transplants with at least one rejection episode survive three years less than those that never experience a rejection episode [23]. The key to protecting corneal transplants is the early and accurate diagnosis of rejection and the prompt initiation of treatment to prevent irreversible damage to the graft [13, 32]. The diagnostic techniques currently available are flawed in this regard [13]. There is insufficient sensitivity and specificity in the tests to detect early rejection, which would enable clinicians to accurately determine the immunological status of the graft and predict its survival [13].

While the tools for *in vivo* studies must be improved in terms of their image capturing and processing systems, the field of pathology is experiencing a real technological revolution with the use of digital microscopy. Articles published in recent years have stated that digital microscopy has many advantages, including the relatively low maintenance cost, the reduced economic barriers related to laboratory physical space and time, and increased access to training materials [33–36]. These advantages were the primary reasons for using digital microscopy in our research.

Despite all of these well-documented advantages of digital microscopy, it has some limitations: it is intrinsically dependent on the proper functioning of computers, software, data storage units, networks, and servers, with variable costs [33]. Due to limitations in local network systems or restricted internet access, the adoption of digital microscopy might not be cost-effective or attainable for some users [33].

Most software automatically selects the area to be scanned and avoids scanning of blank areas without tissues [37]. This automation can, however, cause problems, especially if the slides are poorly stained, and tissues such as fatty tissue might not be automatically recognized [37]. Image focus is another delicate point to highlight during the scanning process [37]. Currently, most commercial slide scanners do not have dynamic focus adjustment during the scanning process. Instead, they use a restricted number of focal points on the slide, often leading to a loss of focus between these areas [37]. In addition, areas with a suboptimal focus, such as tissue folds and air bubbles and incomplete coverage by the coverslip strongly influence the focus of adjacent tissue [37, 38]. Newer scanners, such as that used in the present study, are able to perform continuous focusing during scanning, reducing focus problems [37, 39].

Altogether, the ideal research model to assess the rejection potential of a sample would be to study the donor button before transplanting it, documenting the thickness of its DM and following it throughout its survival as a graft, reporting possible episodes of immune-mediated reactions, and correlating them with the thickness of the DM and age. The corneal lamellae should be studied using noncontact instruments and high-accuracy devices both *in vivo* and *ex vivo*.

Despite the limitations, this study was one of the first to use digital microscopy for the documentation and measurement of corneal layers, comparing samples of pathological tissues with normal tissues; and the applied measurement protocol may be useful for further research studies.

## Figures and Tables

**Table 1:** Comparison of the ages, sides, and measurements of the layers obtained by digital microscopy between normal corneas and corneas from failed grafts due to rejection.

	Normal corneas (*n*=25)	Corneas from failed grafts due to rejection (*n*=40)	*p* value
Age (years, mean ± DP)	81.8 (±6.0)	66.2 (±14.6)	**<0.001**
Side			
Right (%)	13 (52.0)	20 (50.0)	0.875
Left (%)	12 (48.0)	20 (50.0)	
Mean corneal thickness			
Epithelium (*μ*m, 40x)			
Central (mean ± SD)	32.207 (±7.165)	47.683 (±15.518)	**<0.001**
Nasal (mean ± SD)	44.091 (±7.832)	48.228 (±21.514)	0.360
Temporal (mean ± SD)	43.228 (±10.802)	48.918 (±20.797)	0.211
Stroma (mm, 10x)			
Central (mean ± SD)	0.616 (±0.075)	0.561 (±0.163)	0.831
Nasal (mean ± SD)	0.911 (±0.119)	0.588 (±0.175)	**<0.001**
Temporal (mean ± SD)	0.964 (±0.143)	0.607 (±0.162)	**<0.001**
Descemet's membrane (*μ*m, 40x)			
Central (mean ± SD)	8.410 (±2.016)	8.545 (±2.712)	0.820
Nasal (mean ± SD)	14.363 (±4.885)	9.883 (±3.186)	**<0.001**
Temporal (mean ± SD)	13.337 (±3.184)	9.506 (±2.625)	**<0.001**

*n* = number of cases; SD = standard deviation; mm = millimeter; *μ*m = micrometer; 10x, 40x = microscopemagnification. The Kolmogorov–Smirnov test was used to test the equality of probability distribution, whereas the comparisons of two independent variables were performed using Student's *t*-test for continuous variables and the chi-squared test for categorical variables. Levene's test was used to analyze homogeneity.

**Table 2:** Comparison of the mean thicknesses of the Descemet's membrane obtained by digital microscopy in the different regions of normal corneas and corneas from failed grafts due to rejection.

Mean thickness of the Descemet's membrane (*μ*m, 40x)				
	Central	Nasal	Temporal	*p* value

Normal corneas (mean ± SD)	8.410^a^ (±2.016)	14.363^b^ (±4.885)	13.337^b^ (±3.484)	**<0.001**
Corneas from failed grafts due to rejection (mean ± SD)	8.545^a^ (±2.712)	9.883^a^ (±3.186)	9.506^a^ (±2.625)	0.101

*n* = number of cases; SD = standard deviation; *μ*m = micrometer; 40x = microscope magnification; ^a,b^ = letters to identify the differences between the groups in the central, nasal, and temporal measurements of normal corneas and corneas from failed grafts due to rejection. The Kolmogorov–Smirnov test was used to test the equality of probability distribution, and ANOVA and Tukey's test were used for comparisons between multiple variables of the samples.

**Table 3:** Internal validation: comparison between corneal measurements obtained from manual microscopy only and digital microscopy on 10 normal corneas stained with periodic acid-Schiff (PAS).

Corneal measurements	Manual microscopy	Digital microscopy	% Difference
CASE 1			
Total cornea (*μ*m, 10x)	560.00	560.00	0.00
Epithelium (*μ*m, 40x)	23.00	22.91	−0.39
Descemet's membrane (*μ*m, 40x)	7.50	7.50	0.00
CASE 2			
Total cornea (*μ*m, 10x)	570.00	570.00	0.00
Epithelium (*μ*m, 40x)	38.75	38.78	0.08
Descemet's membrane (*μ*m, 40x)	5.27	5.27	0.00
CASE 3			
Total cornea (*μ*m, 10x)	590.00	590.00	0.00
Epithelium (*μ*m, 40x)	48.50	48.49	−0.02
Descemet's membrane (*μ*m, 40x)	10.00	10.04	0.40
CASE 4			
Total cornea (*μ*m, 10x)	690.00	690.00	0.00
Epithelium (*μ*m, 40x)	32.25	32.25	0.00
Descemet's membrane (*μ*m, 40x)	9.25	9.25	0.00
CASE 5			
Total cornea (*μ*m, 10x)	680.00	680.00	0.00
Epithelium (*μ*m, 40x)	36.30	36.31	0.03
Descemet's membrane (*μ*m, 40x)	10.20	10.21	0.10
CASE 6			
Total cornea (*μ*m, 10x)	660.00	660.00	0.00
Epithelium (*μ*m, 40x)	41.80	41.86	0.14
Descemet's membrane (*μ*m, 40x)	8.30	8.31	0.12
CASE 7			
Total cornea (*μ*m, 10x)	520.00	520.00	0.00
Epithelium (*μ*m, 40x)	41.40	41.44	0.10
Descemet's membrane (*μ*m, 40x)	5.60	5.61	0.18
CASE 8			
Total cornea (*μ*m, 10x)	560.00	560.00	0.00
Epithelium (*μ*m, 40x)	38.60	38.59	−0.03
Descemet's membrane (*μ*m, 40x)	7.61	7.61	0.00
CASE 9			
Total cornea (*μ*m, 10x)	620.00	620.00	0.00
Epithelium (*μ*m, 40x)	35.33	35.34	0.03
Descemet's membrane (*μ*m, 40x)	7.26	7.26	0.00
CASE 10			
Total cornea (*μ*m, 10x)	700.00	700.00	0.00
Epithelium (*μ*m, 40x)	30.01	30.03	0.07
Descemet's membrane (*μ*m, 40x)	9.58	9.58	0.00
MEAN OF 10 CASES			*p* value
Cornea total (*μ*m, 10x, mean ± SD)	615.000 (±64.000)	619.000 (±64.000)	0.885
Epithelium (*μ*m, 40x, mean ± SD)	37.295 (±5.802)	36.600 (±7.090)	0.813
Descemet's membrane (*μ*m, 40x, mean ± SD)	8.265 (±1.786)	8.066 (±1.737)	0.804

*μ*m = Micrometer; 10x, 40x = Microscope magnification; SD = Standard deviation. The Kolmogorov–Smirnov test was used to test the equality of probability distribution, whereas the comparisons of two independent variables were performed using Student's *t*-test for continuous variables. Levene's test was used to analyze homogeneity.

**Table 4:** Internal validation: comparison between corneal measurements obtained by manual microscopy only and by digital microscopy on 10 corneas from failed grafts stained with periodic acid-Schiff (PAS).

Corneal measurements	Manual microscopy	Digital microscopy	% Difference
Case 1 (corneal opacities with 01 previous PK rejected)			
Total cornea (*μ*m, 10x)	520.00	520.00	0.00
Epithelium (*μ*m, 40x)	5.50	5.51	0.18
Descemet's membrane (*μ*m, 40x)	7.80	7.70	−1.28
Case 2 (corneal opacities with 01 previous PK rejected)			
Total cornea (*μ*m, 10x)	280.00	280.00	0.00
Epithelium (*μ*m, 40x)	20.80	20.87	0.34
Descemet's membrane (*μ*m, 40x)	4.00	4.07	1.75
Case 3 (Fuchs distrophy with 01 previous PK rejected)			
Total cornea (*μ*m, 10x)	370.00	370.00	0.00
Epithelium (*μ*m, 40x)	24.60	24.62	0.08
Descemet's membrane (*μ*m, 40x)	7.60	7.63	0.39
Case 4 (pseudophakic bullous keratopathy with 01 previous PK rejected)			
Total cornea (*μ*m, 10x)	560.00	560.00	0.00
Epithelium (*μ*m, 40x)	50.10	50.16	0.12
Descemet's membrane (*μ*m, 40x)	10.60	10.61	0.09
Case 5 (pseudophakic bullous keratopathy with 01 previous PK rejected)			
Total cornea (*μ*m, 10x)	610.00	610.00	0.00
Epithelium (*μ*m, 40x)	37.70	37.66	−0.11
Descemet's membrane (*μ*m, 40x)	8.90	8.93	0.34
Case 6 (keratoconus with 01 previous PK rejected)			
Total cornea (*μ*m, 10x)	410.00	410.00	0.00
Epithelium (*μ*m, 40x)	63.70	63.73	0.05
Descemet's membrane (*μ*m, 40x)	4.00	4.03	0.75
Case 7 (pseudophakic bullous keratopathy with 01 previous PK rejected)			
Total cornea (*μ*m, 10x)	910.00	910.00	0.00
Epithelium (*μ*m, 40x)	52.40	52.40	0.00
Descemet's membrane (*μ*m, 40x)	7.90	7.90	0.00
Case 8 (corneal opacities with 01 previous DSAEK rejected)			
Total cornea (*μ*m, 10x)	570.00	570.00	0.00
Epithelium (*μ*m, 40x)	41.30	41.30	0.00
Descemet's membrane (*μ*m, 40x)	7.20	7.26	0.83
Case 9 (keratoconus with 01 previous PK rejected)			
Total cornea (*μ*m, 10x)	700.00	710.00	1.43
Epithelium (*μ*m, 40x)	52.85	52.91	0.11
Descemet's membrane (*μ*m, 40x)	13.80	13.86	0.43
Case 10 (keratoconus with 01 previous PK rejected)			
Total cornea (*μ*m, 10x)	390.00	390.00	0.00
Epithelium (*μ*m, 40x)	72.40	72.48	0.11
Descemet's membrane (*μ*m, 40x)	4.00	4.07	1.75
Mean of 10 cases			*p* value
Cornea total (*μ*m, 10x, mean ± SD)	532.000 (±184.000)	541.000 (±185.000)	0.918
Epithelium (*μ*m, 40x, mean ± SD)	42.140 (±20.523)	42.164 (±20.532)	0.998
Descemet's membrane (*μ*m, 40x, mean ± SD)	7.580 (±3.132)	7.545 (±3.129)	0.980

*μ*m = micrometer; 10x, 40x = microscope magnification; PK = penetrating keratoplasty; DSAEK = automated endothelial keratoplasty; SD = standard deviation. The Kolmogorov–Smirnov test was used to test the equality of probability distribution, whereas the comparisons of two independent variables were performed using Student's *t*-test for continuous variables. Levene's test was used to analyze homogeneity.

## Data Availability

All data can be accessed at MUHC-McGill University Ocular Pathology & Translational Research Laboratory (Montreal, Canada).
